# Intestinal Pseudo-Obstruction as the Initial Clinical Presentation in Systemic Lupus Erythematosus: A Rare and Severe Disorder

**DOI:** 10.1155/2020/8873917

**Published:** 2020-11-16

**Authors:** Akwe Nyabera, Mohanad Elfishawi, Francisco Cuevas, Fahad Riaz, Adriana Abrudescu

**Affiliations:** NYC Health + Hospitals/ Queens, Jamaica, NY, USA

## Abstract

Intestinal pseudo-obstruction (IPO) is a rarely recognized complication of systemic lupus erythematosus (SLE). We present a 36-year-old African American female, with only known past medical history of anemia, admitted for frequent vomiting, abdominal distension, abdominal pain, diarrhea, and fever that had been ongoing for 5 days. Laboratory results revealed leukopenia and thrombocytopenia. Imaging revealed dilated small bowel loops, abdominal ascites, as well as mild bilateral hydroureteronephrosis without obstructing calculus. Serologic testing confirmed a diagnosis of SLE. The patient was placed on immunosuppressive therapy and responded well. IPO has previously been described as a rare finding in patients with SLE, with bilateral hydroureteronephrosis and lupus interstitial cystitis having been noted as common concomitant factors. One must have a high level of suspicion to recognize it as being one of the initial clinical presentations. Early recognition and appropriate management preclude unnecessary invasive procedures that do not take into account the pathophysiology of the condition and allow for appropriate management and return of peristaltic function.

## 1. Introduction

Systemic lupus erythematosus-related intestinal pseudo-obstruction was initially reported in the 1970s [1]. It is more common in Asian populations and usually presents with signs and symptoms of large or small bowel obstruction in the absence of mechanical obstruction. This results in decreased flow of abdominal contents and may be complicated by perforation, ischemia, and infarction [1, 2]. IPO is only seen in 1.96% of patients with SLE. Of these 1.96% patients, only approximately 57.6% present with it as the initial manifestation [2]. The pathophysiology of IPO in SLE remains unclear; however, one possible mechanism includes systemic autoimmune processes targeting smooth muscle cells, supported by the high concomitant lupus cystitis and hydroureteronephrosis, with the latter witnessed in up to 63.6% of cases [3, 4]. Other theories that have been postulated include vasculitis leading to chronic bowel ischemia as well as intrinsic muscle dysmotility affecting the muscularis propria [4, 5]. An understanding of the pathophysiology and appropriate treatment is vital for favorable outcomes.

## 2. Case Report

A 36-year-old African American female presented with abdominal distension, diarrhea, nausea, and vomiting that had been ongoing for a week. Her past medical history was only significant for anemia. However, when questioned further, she also described a history of ankle pain and a dark rash behind her ears that had been present for 3 weeks. She had initially presented to the ED with similar complaints two days prior to admission and was discharged home with metronidazole and ciprofloxacin under the impression of bacterial gastroenteritis. However, she returned when her symptoms progressively worsened.

**Figure 1:**
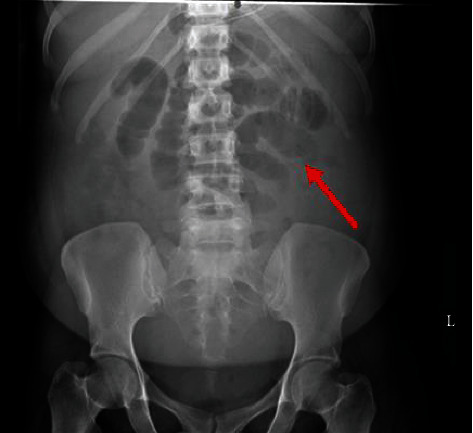
2-view abdominal X-ray showing dilated loops of small bowel.

**Figure 2:**
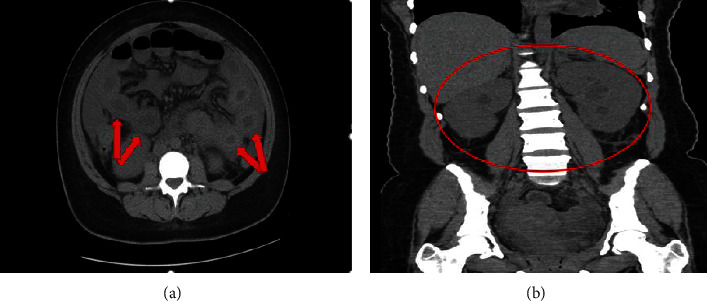
Abdominal CT scan showing small bowel thickening (a), bilateral hydroureteronephrosis (b), and moderate intra-abdominal ascites.

Physical examination revealed a distended abdomen with normoactive bowel sounds that was nontender to palpation. Laboratory results revealed elevated acute phase reactants, anemia, and acute renal insufficiency. A 2-view abdominal X-ray showed dilated loops of small bowel suggestive of small bowel obstruction (Figure 1); however, there was no obvious transition point or visible obstruction. Computed tomography (CT) scan findings showed small bowel thickening and were also notable for bilateral hydroureteronephrosis and moderate intra-abdominal ascites (Figures 2(a), 2(b)). Initial treatment was targeted at IPO in the setting of enteritis, and she was treated conservatively via nasogastric tube insertion, administration of antibiotics: vancomycin, meropenem, and doxycycline, as well as nil per os (NPO) status.

Her kidney function continued to worsen with an increase in her creatinine level from a baseline of 0.75 mg/dL to 4.55 mg/dL. Urinalysis was negative for blood and protein. CT scans showed persistent bilateral ureter hydronephrosis, and renal Lasix scan revealed decreased renal flow and function bilaterally, with no obstruction in either kidney. Given the fact that the urinalysis was negative for blood and protein, the observed acute renal failure was determined to be less likely due to lupus nephritis and more likely to be secondary to the bilateral hydroureteronephrosis, frequently associated with lupus interstitial cystitis. Another factor taken into consideration was her reduced oral intake and frequent vomiting which may have contributed to the acute kidney injury. Insertion of bilateral ureteral stents for the hydroureteronephrosis was discussed; however, due to the lack of visible obstruction, the procedure was foregone.

Further rheumatologic workup showed elevated antinuclear antibody (1 : 1280), double-stranded DNA antibody (>1000 IU/mL), anti-Smith antibody (>8 AI), erythrocyte sedimentation rate (ESR) (106 mm/hr) and C-reactive protein (CRP) (51.4 mg/L), low C3 complement (27 mg/dL) and low C4 complement (4 mg/dL), as well as positive direct antiglobulin test IGG, positive anti-cardiolipin antibody, negative anti-beta-2-glycoprotein I antibody, negative lupus anticoagulant, negative rheumatoid factor, and negative SSA and SSB. Total WBC count was (3.67 K/*µ*L) with decreased absolute lymphocyte count of (0.72 K/*µ*L). Additionally, persistent thrombocytopenia was also observed, with a platelet cell count drop to (44 K/*µ*L), indicating lupus thrombocytopenia and involvement of multiple systems.

**Figure 3:**
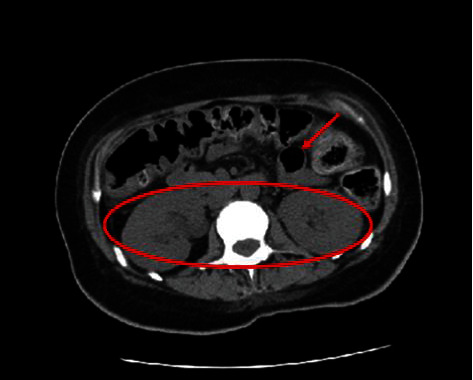
Resolution of previously seen hydronephrosis (red circle), dilated loops of bowel (red arrow), and ascites.

The patient was treated with intravenous methylprednisolone 1 gm daily for 5 days and continued on maintenance steroid therapy which was slowly tapered and switched to oral methylprednisolone. She also received 5 days of intravenous immunoglobulin therapy (IVIG) at 400 mg/kg/day for persistent thrombocytopenia, with an improvement in her platelet count. This, in addition to aggressive electrolyte repletion, enteral nutrition, and antibiotics, showed a notable improvement in her IPO, renal failure, and ascites, with marked changes noted on repeat CT scan performed prior to discharge (Figure 3).

The patient was then discharged on oral methylprednisolone with follow-up in the rheumatology clinic and later started on hydroxychloroquine 200 mg daily twice daily and mycophenolate mofetil (CellCept) 1 gm twice daily as steroid-sparing agent. SLE workup at the six-month clinic visit revealed normal levels of Smith antibody (0.5 AI), C3 complement (111 mg/dL), and C4 complement (33 mg/dL) and negative dsDNA antibody. All her bowel symptoms fully resolved and did not recur.

## 3. Discussion

Gastrointestinal manifestations such as esophageal hypomotility, malabsorption, mesenteric vasculitis, and pancreatitis are common in patients with SLE [6]. We present a rare case of IPO as one of the initial symptoms of active SLE. In an 11-year longitudinal study conducted by Zhang et al, they found that, amongst 4331 patients with SLE, IPO was present in 1.96%, with an in-hospital fatality rate of 7.1%. IPO was the initial presenting factor in approximately 57.6% of these patients with a rate of misdiagnosis of 78%. Another two studies that took into account both newly diagnosed SLE and previously known cases indicated that IPO was the initial presenting symptom 50% and 41% of the time [2, 5].

Signs and symptoms include abdominal distension, diarrhea, nausea, vomiting, weight loss, or constipation. From a review of the literature, diagnosis of lupus after manifestation of symptoms can range from anywhere between 2 and 25 days in active SLE and up to 3 years in inactive SLE [7]. Laboratory tests are often helpful in the diagnosis of SLE. In the study by Zhang et al. they found that, when compared, laboratory findings revealed that positive ANA, elevated CRP, hypoalbuminemia, hypocomplementemia, positive anti-SSA antibodies, and positive anti-SSB antibodies were more common in patients with IPO-related SLE [2]. Our patient's laboratory results were significant for a high titer antinuclear antibody (1 : 1280), double-stranded DNA antibody (>1000 IU/mL), and anti-Smith antibody (>8 AI).

Several imaging modalities such as abdominal X-ray, CT scan, voiding cystourethrogram, and renal Lasix scan may be used to provide further insight into the condition. On imaging, our patient was noted to have diffusely dilated viscera, small bowel thickening, abdominal ascites, and concomitant bilateral hydroureteronephrosis in the absence of any identifiable structural obstruction. Hydroureteronephrosis is a finding that has been noted in up to two-thirds of patients with IPO-related SLE, and more rarely, in some cases, hepatobiliary involvement in the form of megacholedochus has also been observed [2, 4, 7].

The pathogenic mechanism remains unclear; however, several theories have been put forth in the literature. The involvement of the gastrointestinal, hepatobiliary, and genitourinary systems suggests possible associated smooth muscle dysmotility secondary to vasculitis and immune complex deposition in the intestinal tract and bladder vessels [3, 8, 9]. Common autoantigens in the gastrointestinal wall and bladder may be responsible. A strong link between hydroureteronephrosis, interstitial cystitis, and intestinal pseudo-obstruction has previously been observed [3, 9]. Indeed lupus interstitial cystitis is associated with hydroureteronephrosis in up to 92% of cases [10]. This is because of distal ureteral obstruction at the ureterovesical junction secondary to bladder inflammation and edema, as well as detrusor muscle spasm in the setting of inflammation [11]. Histological examination reveals edema and inflammation of the submucosa associated with a high amount of eosinophilic infiltration. Necrosis and loss of myocytes in the muscularis propria and atrophy of the mucosa is also observed. A few studies have found abnormalities of innervation and occasional vasculitis in the absence of thrombosis [4, 7, 8, 12].

As with our patient, medical management of IPO-related SLE is attained with immunosuppressive therapy combined with corticosteroids. Prognosis of the disease often depends on the presence of vital organ involvement [2, 13]. Depending on the severity of the symptoms, high-dose IV pulse corticosteroids can be used initially, switched to oral therapy, and then tapered off gradually. These measures in combination with adequate supportive care (oral antibiotics, parenteral nutrition, and adequate hydration) often result in a favorable prognosis, with the resolution of the IPO and urinary symptoms [4, 5]. It is important to note that, on review of the literature, several studies describe patients undergoing surgical intervention, such as exploratory laparotomy and insertion of ureteral stents; however, these procedures provide no additional benefits, are unnecessary, and put the patient at an increased risk for complications [2, 4, 7, 13].

In conclusion, IPO has been recognized as a rare complication of SLE. It commonly occurs with associated urinary tract involvement, and a high clinical suspicion is required to diagnose it. Early diagnosis confers a more favorable prognosis, allowing for appropriate medical management and avoidance of unnecessary surgical intervention.

## Data Availability

The data used to support the findings of this study are available from the corresponding author upon request.
